# Effect of Hopper Loading on the Formation of Alkyl Alcohols in Olive Fruits and Its Relationship with Sensory Quality Losses of Virgin Olive Oil

**DOI:** 10.3390/foods12132633

**Published:** 2023-07-07

**Authors:** Abdelaziz Boudebouz, Agustí Romero, Juan-F. Hermoso, Ricard Boqué, Montserrat Mestres

**Affiliations:** 1 Department of Analytical Chemistry and Organic Chemistry, Universitat Rovira i Virgili (URV)—Chemometrics and Sensorics for Analytical Solutions Group (ChemoSens), Campus Sescelades, 43007 Tarragona, Spain; 2 Institut de Recerca i Tecnologia Agroalimentària (IRTA-Mas de Bover), Ctra Reus-El Morell Km 3.8, 43120 Constantí, Spain

**Keywords:** premium olive oil, olives, hopper, short-term storage, alcohols, quality

## Abstract

The storage of olives in large hoppers is a widespread practice in oil mills, but these large volumes and their unloading can cause a physical deterioration of the olives that will affect the quality of the oil obtained. This research deals with the effect of hopper charge on the formation of alkyl alcohols in olive fruits and its relationship with the sensory quality losses of ‘Arbequina’ virgin olive oil. The contents of ethanol, methanol, and acetaldehyde were measured in olive samples loaded and stored for a short time in a large hopper and analyzed at three different hopper-discharging times, which are related to three different positions inside the hopper. The corresponding oil from each sampling was obtained by using ABENCOR and was evaluated by a trained tasting panel. Results showed that the ethanol content in olives increased during their storage in the hopper, while methanol and acetaldehyde contents did not show significant differences. Regarding their position in the hopper, fruits located at the bottom or on the lateral sides showed a greater deterioration. The sensory analyses showed an inverse relationship between the positive attributes of olive oils and their content of alcohols.

## 1. Introduction

The olive oil quality directly depends on the fruit characteristics and its quality status before the milling process. It is influenced by many agronomic, harvesting, and processing factors [1]. Control over the different production steps is crucial; but since they are not independent of each other, it is necessary to ensure that the transition from one step to another is also controlled. To prevent the physical and biological deterioration of the olive fruits between harvest and processing, some strategies have been proposed, such as the transport and storage of olives in perforated boxes of less than 1000 kg capacity or keeping them at low temperatures until processing [2,3,4]. In bigger industries, the current practices are based on the reduction of the time elapsed between the harvest and the milling process and the main recommendation is to increase the milling capacity to process the olives in a period of less than 24 h [5] or even below 12 h when the milling characteristics allow it [6]. However, when this is not possible and the volume of fruit exceeds the capacity of the mill, the olives are stored for a longer period in large-capacity hoppers, which can become a serious problem.

In Spain, most of the large olive oil mills receive a quantity of olives greater than the milling capacity. This problem requires the use of hoppers with a capacity from 20 to 40 tons where the fruits are stored until they can be processed. As for medium or small mills, these also use large hoppers to minimize the space required in the building. Under these conditions, the fruits located at the bottom of the hopper are subjected to greater pressure and, when the olives are very ripe, there is a risk that they may even be squeezed [7]. It is assumed that the olives situated at the bottom of the hopper are the only ones subjected to quality deterioration, while the rest of the fruits inside the hopper maintain their quality but, to date, no experimental results are available to confirm this statement. In fact, the only results related to this topic are those reported by Vichi et al. [8], who found a significant increase in the winey and musty defects and volatile phenols during fruit storage in closed plastic bags. They concluded that the ‘Arbequina’ variety is more susceptible to postharvest damages than other varieties such as ‘Arbosana’ or ‘Leccino’. These results are especially relevant because ‘Arbequina’ is the most widely cultivated variety in the new super-high-density orchards worldwide. The olive fruits from these orchards are mechanically harvested and delivered to the mills in large quantities, so it is necessary for their storage in hoppers until they are processed. Therefore, considering the high susceptibility to damage of the ‘Arbequina’ variety, this way of proceeding becomes a handicap to producing high-quality oils because, during this storage period, the olives are subjected to pressure and heat, which provide an optimal medium for the growth of fungi and bacteria [8,9]. Moreover, the deterioration of the olives during storage caused by both aerobic and anaerobic processes increases the acidity of the oil [10] and promotes the production of alcohols and other compounds [6], with the subsequent sensory impact on the final product. 

The sensory quality is a crucial parameter in olive oil because it conditions the classification of the oil into different categories. Thus, extra virgin olive oil (EVOO), the highest quality olive oil with highly beneficial effects on health, must have some positive sensory flavors and no sensory defects [11,12]. Within this group, there is a new trend focused on the production and labeling of the so-called “premium” extra virgin olive oils. These oils present a fresh and complex flavor with high values of harmony quality factor since the aromas coming from the lipoxygenase pathway have developed properly and remain well balanced in the oil and without anomalous aromas that can overlap them [12,13]. Hence, while it is important to promote the formation of pleasant aromas, it is even more important to avoid the formation of unpleasant ones taking into account that an aroma is often due to the combination of the odor of different volatile compounds. This is the case of some sensory olive oil defects that are related to the fruit quality, such as winey–vinegary, fusty, muddy, and musty [14,15,16,17,18,19]. 

Although monitoring the generation of these off-flavors would be very interesting for the quality control of virgin olive oil, the legislation does not set limits for these compounds. However, it does limit the amount of fatty acid alkyl esters (FAAEs), which originate from the esterification of free fatty acids with short-chain alcohols (mainly ethanol and methanol) [20,21,22]. Consequently, to comply with the legislated maximum content of alkyl esters (35 mg/kg for ethyl esters), it is necessary to avoid the formation of these precursors throughout the entire production process, starting with the control of the quality of the olives, their storage, and the extraction conditions [23,24]. In addition, the fact that ethanol and methanol are involved in both sensory defects and FAAEs formation makes them good markers for checking the possible adverse effects.

The objective of this study was to evaluate how short-term storage in a large-capacity hopper affects the formation of alkyl alcohols in olives and its influence on the presence of sensory defects in the olive oil obtained. The results will expand the knowledge about the production conditions that allow for obtaining a premium EVOO. 

## 2. Materials and Methods

### 2.1. Experiments

The experiments were carried out with olives of the ‘Arbequina’ variety from the 2018 olive campaign and working under real conditions in the mill of the Cooperative of La Granadella (Catalonia, Spain) which operated according to the usual processing method of this geographical zone to obtain high-quality EVOO. The mill hopper had a capacity of 25,000 kg of fruit (with dimensions of 4 m × 4 m × 2.5 m); it was made of stainless steel and equipped with a vibrating system to outflow the olives from the bottom.

To obtain reliable results, the experiments were repeated at three different moments of the 2018 olive campaign: beginning, midseason, and near the end (i.e., 26 Nov., 12 Dec., and 7 Jan.). In this way, three different states of olive ripeness were achieved (green, ripe, and overripe, respectively). On each date, 22,000 kg of olives were harvested, delivered, uploaded, and processed on the same day. This way of working meant that the olives remained in the hopper for only three hours (that is, short-time storage) until they were discharged. To evaluate the effect of the hopper, olive samples were taken at different discharging times.

### 2.2. Sampling

Olive samples were taken and their temperature was measured while the hopper was unloading (just as the olives were falling from the bottom of the hopper) at three different times: 1 min (T1), 10 min (T10), and 20 min (T20). At each time, a representative and homogenous sample of olive was taken in triplicate in perforated boxes of 3 kg. The outflow rate was 4000 kg per hour and the sampling times are equivalent to the times that the olives remain in the hopper depending on the height at which they are inside the hopper (T1 at the base of the hopper, T10 in the middle of the hopper, and T20 at the top of the hopper). 

The olive samples were transferred directly to the laboratory and each sample was split into three parts. 

#### 2.2.1. Determination of Physicochemical Properties

The first part was used to determine the fruit characteristics: maturity index [25], health status, fruit weight, flesh/pit weight ratio (*w*/*w*), moisture, and oil content. The health status of the fruits was determined by a visual diagnosis of 100 olives randomly taken; the bruised, the smashed, the broken, and the fermented fruits were classified as damaged fruits. The results were expressed as a percentage of healthy and damaged fruits.

The moisture content of the olive fruits was measured gravimetrically after drying them at 105 °C for 24 h. The oil content was determined by using the Soxhlet [26] method. Both values were expressed as a percentage of the total weight of the sample.

#### 2.2.2. Sample Preparation for Subsequent GC Analysis

The second part was used to determine the ethanol and methanol content in olive fruit homogenates, following the method described by Boudebouz et al. [27]. Briefly, 100 g of olives were crushed and well homogenized, and then 15 g of the olive paste were weighed in a 50 mL falcon tube together with 15 g of MilliQ water and mixed well using a vortex stirrer. Two grams of this homogenate were weighed in a 20 mL vial together with two grams of a saturated solution of CaCl_2_ in water (10%) and were kept in a freezer (at −18 °C) until its analysis by headspace solid-phase microextraction gas chromatography (HS-SPME GC-MS). 

#### 2.2.3. Obtaining Olive Oils

Finally, the third part of the olives was processed using the ABENCOR system to obtain a representative oil from each sample. The olives were crushed and the olive paste was malaxed at 24 °C for 30 min and then centrifuged to obtain the oily fraction. In order to clarify the oil obtained in the previous step, a second high-speed desktop centrifuge (KUBOTA Model, Osaka, Japan) was used. The oil samples obtained were filtered to remove the residual impurities and sent to the tasting panel for sensorial analysis.

#### 2.2.4. Reference Samples

To evaluate the effect of the hopper on the olive fruits, the results of each sampling were compared with those belonging to the so-called reference olive samples (Ref). To obtain them, small quantities of olives were taken from each of the different batches as soon as they arrived at the oil mill by using the scale’s aspirator-sampler. These Ref samples were stored under ambient conditions, next to the hopper and in small perforated boxes of 3 kg until the end of the experiment. This sampling was carried out throughout the entire hopper loading process to ensure a representative reference sample of all partial entries of olives into the hopper. 

### 2.3. Chemicals and Reagents

The standards of ethanol absolute (gradient HPLC grade) and methanol (supragradient HPLC grade) were purchased from Scharlab (Barcelona, Spain). Acetaldehyde standard (99% for synthesis) and calcium chloride anhydrous (97%) were purchased from Panreac (Barcelona, Spain). The Milli-Q quality water used was obtained from a laboratory purification system (Millipore, Bedford, MA, USA).

For the Headspace-Solid Phase Microextraction (HS-SPME) of the analytes, 2 cm length fibers 50/30 μm StableFlex divinylbenzene/carboxen/polydimethylsiloxane (DVB/CAR/PDMS) were purchased from SUPELCO (North Harrison Road, Bellefonte, PA, USA) were used.

### 2.4. Analytical Procedure 

The quantitative determination of the analytes (ethanol, methanol, and acetaldehyde) in each sample was done in triplicate by using the method previously optimized [27]. It consists of applying the solid phase microextraction technique to the headspace of the samples (HS-SPME) with subsequent analysis of the extract by gas chromatography coupled to a quadrupole mass spectrometer (GC-MS). 

Specifically, the vials containing the homogenates of the samples were preconditioned for 5 min at 40 °C and, afterward, the SPME fiber was inserted through the vial septum and exposed to the headspace above the sample for 50 min at 40 °C with medium orbital agitation. Then, the fiber was removed from the vial and introduced directly into the injector port of the GC-MS for thermal desorption at 270 °C for 1 min in splitless mode.

The chromatographic analyses were performed with an HP-6890 gas chromatograph (HP, Palo Alto, CA, USA) equipped with an HP-5973 mass selective detector (HP, Palo Alto, CA, USA). The chromatographic separations were carried out using a fused silica capillary column Chrompack, CP-WAX 57CB (50 m × 0.25 mm i.d. and 0.2 μm film thickness) (Varian, Middelburg, The Netherlands) with an oven temperature program of 40 °C (5 min), 5 °C min^−1^ to 100 °C, and 10 °C min^−1^ to 215 °C (5 min). The carrier gas was helium (He), with a head pressure of 14.8 psi at a constant flow of 1.8 mL min^−1^. The mass spectrometer was operated in the electron impact ionization mode at 70 eV. Ion source, mass quadrupole, and interface temperatures were 200 °C, 230 °C, and 150 °C, respectively. The mass-to-charge (*m*/*z*) ratio range used was 28–300 amu. The identification of the compounds was done by spectrum matching with the Wiley/NBS library. 

For the quantification of the analytes studied, the calibration lines were built using the matrix-matched calibration technique. As explained in previous studies [27], this technique avoids quantification errors due to the matrix effect. The calibration standards ranged between 0–200 mg kg^−1^ for both ethanol and methanol and between 0–15 mg kg^−1^ for acetaldehyde. The regression lines showed determination coefficients R^2^ > 0.96 in all cases.

### 2.5. Sensory Evaluation

The sensory evaluation of the different olive oil samples was carried out by the Official Tasting Panel of Virgin Olive Oils of Catalonia (Reus, Spain), which has been recognized by the IOC since 1997 and by the Spanish Government since 2004. The panel follows the ISO 17025 standard since 2002 and uses the official scoring sheet described by the official method. Each sensorial attribute was measured by eight trained tasters using a 10 cm open scale anchored to zero. The median of the eight tasters was used to describe the final intensity of each attribute. Since the planned experiments involved short periods of time to generate changes, only slight sensory variations were expected. To better detect the possible differences and obtain a global comparison between the samples, two new descriptors were also evaluated [28,29]. These descriptors are called “complexity” and “global sensory score, and both were calculated based on the results obtained in the evaluation of the official descriptors provided by the eight tasters. The “complexity” descriptor is defined as the number of secondary aromas detected by more than 30% of tasters, which gives an idea of how many aromas the oil has, besides those expected [12]. Regarding the “global sensory score”, it facilitates the comparison of the sensory quality of different samples and its value is calculated using an algorithm developed by Romero et al. [28] ranging from 0 (very poor quality) to 9 (maximum quality). Thus, for example, while the EVOO global sensory score should be at least 6.5 points, when it comes to the premium category, this value should be at least 7.2 [28,30]. 

### 2.6. Statistical Analysis

The effect of the hopper loading was statistically analyzed by ANOVA (Analysis of Variance) with the SAS-Stat Software (V9.4. Cary, SAS Institute Inc., Hong Kong, China), using the generalized linear model (GLM) procedure, and mean comparisons were performed by using Duncan’s multiple range test (α < 0.05). A partial least squares (PLS) regression model was built using the Unscrambler^®^ X software (version 10.5.1, CAMO Software AS, Oslo, Norway) to relate the fruit characteristics and content of alcohols to the sensory attributes.

## 3. Results and Discussion

### 3.1. Fruit Characteristics

The results of the visual diagnosis of the olives at different times of hopper discharging (Table 1) show a significant deterioration in the quality of fruits after passing through the hopper compared to the reference samples. Olives were mainly damaged due to the impacts that occur during the uploading and discharging of the hopper, in agreement with what was pointed out in other studies [1,7,31]. 

Olives entered on 27 November (date of first sampling) showed 12% of the fruits damaged; the maturity index was 2.4 (yellow to reddish) and they were slight ($\bar{x}$ = 1.08 g) with a low flesh to pit ratio ($\bar{x}$ = 4.1 g/g) and a high moisture and oil contents. After remaining in the hopper for 3 h, visual damage of the fruits increased from 12 to 18% and so did the temperature, which raised from 9 to 12 °C, meaning that some microbiological activity happened in the hopper [8]. 

**Figure 1:**
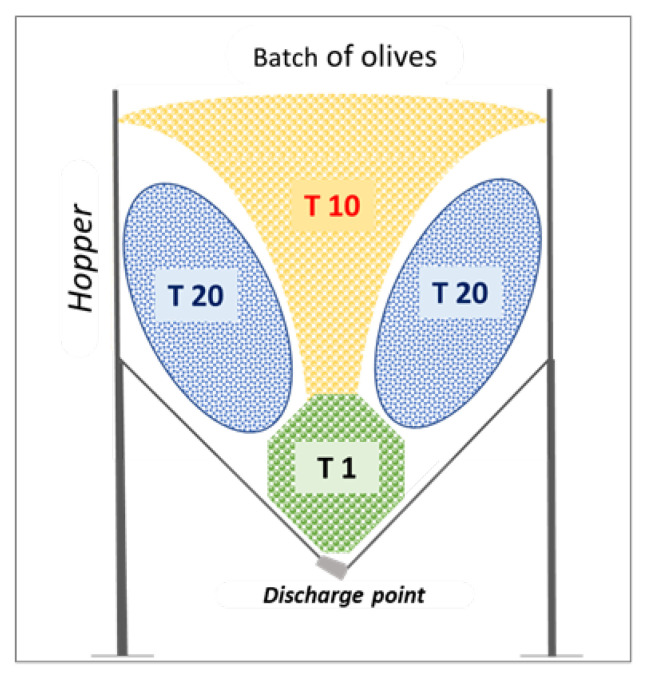
Representative scheme of the sample’s distribution inside the hopper.

Olives delivered by growers on 12 December (date of second sampling) looked very healthy with only 6% of damaged fruits (Table 1); the maturity index was 2.5, and the contents of moisture and fat were very similar to the previous sampling, whereas fruits were heavier ($\bar{x}$ = 1.28 g) and had a higher flesh to pit ratio ($\bar{x}$ = 5.1 g/g). However, after staying 3 h in the hopper, these fruits suffered more damage than in November, raising from 6% to a maximum of 48% while the temperature increased from 7 to 12 °C. An explanation for these differences could be that, in this batch, the fruits were larger and with a higher flesh-to-pit ratio, which could make the fruits softer and more sensitive to the impacts that occur during postharvest. In addition, the samples represented by T20 showed the worst fruit quality. After careful observations, we found that this position corresponds to the olives located on the sides of the hopper and the olives here located are exposed to higher damage by bruises. It should be noted that, in order to relate the time that the olives remained in the hopper with the position of the fruits inside the hopper, we carefully observed the loading and outflow of the hopper, mainly for positions T10 and T20 where we placed a witness and timed the time elapsed until it left the hopper (Figure 1).

Finally, olives entered on 7 January (the date of the third sampling) showed a very significant deterioration due to frost in the orchards. This degradation implies the breakdown of the cell wall that facilitates the fermentation of the olive fruit with the subsequent loss of quality. In fact, up to 40% of the fruits delivered by growers were damaged; they were slight ($\bar{x}$ = 1.03 g), with a maturity index from purple to black (maturity index = 3.5) and a very low flesh-to-pit ratio, implying lower moisture content and, consequently, a higher oil content. After staying 3 h in the hopper, the proportion of damaged fruits increased from 40% to a maximum of 54% while the temperature increased from 5 to 10 °C.

Therefore, the fruits suffered microbiological damage in the hopper regardless of the harvest date and even within three hours. Contrary to what was expected, the olives on the sides of the hopper were the last to be discharged (T20), so they presented the worst quality. This can be explained by the fact that such large hoppers outflow fruits more quickly from the center than from the sides, where the friction with the fruits is greater. Consequently, at the end of the discharging process, the olives on the sides are classified as damaged after visual analysis because they had suffered both the friction with the walls of the hopper and the high pressure of the upper layers.

### 3.2. Tasting Panel Results

As expected, the olive oil sensory quality was related to the quality of the olive fruits. In all experiments, the oils obtained from the reference samples, that is, those that did not pass through the hopper, showed the best sensory profile that could be classified as premium quality, mainly in the first and second experiments (Table 2). Specifically, in the first experiment, all samples produced extra virgin olive oil, although only the reference sample produced a premium extra virgin olive oil [12]. The second experiment resulted in an extra virgin category at T1 and T10 but not at T20, which produced a virgin olive oil with some low-intensity defects. Nevertheless, in the experiment of 7 January, the delivered olives were of very low quality and clearly damaged by freezing, so the oils obtained were categorized as virgin at T10 and T20 and as lampante at T1. In agreement with this classification, the tasters identified the frost defect in the oil extracted from these olives (Table 2), which was more intense when the olives remained in the hopper for three hours. This could be due to the fact that fruitiness also decreased, so any aromatic defects are perceived as more intense because fruitiness overlaps with the other oil aromas. 

On the other hand, when the olives were stored in the hopper, even for a short time, they suffered a degradative process that resulted in a loss of oil quality [31]. This can be easily verified using the global score (Table 2), which decreased from 7.2 to 5.9 in the first experiment (27 Nov.) and from 7.6 to 5.4 in the second experiment (12 Dec.). In the last experiment, very slight differences were observed because of the low initial quality of the fruits, although the trend was also towards a decrease in quality. 

Regarding the charge distribution in the hopper, the highest pressure and weight are exerted at positions T1 and T20. In fact, when studying the oils obtained from olives located in these positions, although the median of all the defects was equal to zero in the first experiment (27 Nov.), the high standard deviation showed that some tasters detected some kinds of fustiness, mustiness, and winey smells, which may be related to some fermentation processes [18]. In the second experiment (12 Dec.), the worst conditions seem to be the ones at the T20 position because some defects, such as musty, fusty, and winey, with an average intensity value above zero, were found in the oils, which coincide with the highest level of fruits damaged in this position (Table 1). Concerning the musty defect, it must be pointed out that this descriptor does not distinguish between molds and yeasts. In sample T20 from the second trial, it can be observed that bitterness, pungency, and astringency decreased significantly, which could be related to the degradation of some polyphenols (mainly secoiridoids) by yeasts, which, in turn, can release volatile phenols that tasters qualify as musty [8]. All these results show that the T10 position produces a better oil. However, in industrial oil mills, it is not possible to divide the olives contained in the hopper to produce different oils. Therefore, although there are olives in the hopper that produce high-quality oils if they are mixed with poor-quality olives, their defects will spread throughout the entire oil batch with the consequent loss of quality. 

### 3.3. Content of Alcohols

The results of the analysis of the alcohols (ethanol and methanol) show a significant increase in the ethanol content in the fruits left in the hopper, compared to the reference samples (Table 3). Furthermore, the positive sensory attributes detected in the good quality oils (mainly fruity, bitter, and pungent) are negatively correlated with the levels of alcohols and acetaldehyde [32].

On average in the three experiments, the content of ethanol increased from 71.5 mg kg^−1^ at the reception point to 164.7 mg kg^−1^ at the worst point in the hopper (Table 1). This increase in the ethanol content seems to be related to fermentation processes, as corroborated by the increase in the temperature of the olives because of this biological process [32]. Moreover, in the two first experiments, a significant increase in the acetaldehyde content was also detected during storage in the hopper. This could be related to the physiological activity of the fruits together with some uncontrolled microbiological activity [33].

It is worth mentioning that, in the third experiment, no significant differences were observed due to the low initial quality of the fruits; in fact, these fruits were the most affected by frost and the olives already arrived at the mill with high amounts of ethanol (138 mg kg^−1^).

Observing the different positions in the hopper, olives from T20 in the second experiment stand out for their very high ethanol content. This behavior coincides with the highest level of damage detected in the olives for this ripening point since the damaged fruit increased eight times compared with the Ref samples. Furthermore, the T20-DEC sample showed the greatest sensory defects directly related to fruit damage (fusty, musty, and winey). These results corroborate those published in previous works [18,19,34], so it seemed logical to attribute the ethanol content increase to the fruit damage. However, the ethanol contents of T20-JAN (corresponding to the analysis of overripe samples with 54% damaged fruits) do not support this theory because it showed about two times less ethanol content when having a slightly higher damage stage than T20-DEC. This behavior suggested that the type of damage must also be taken into account, in addition to the % of damaged fruits. In this way, while T20-DEC suffered significant damage caused by the hopper, in T20-JAN the damage was mainly due to the freezing of the olives and this different origin resulted in different ethanol contents.

Regarding methanol, the high variability between replicates observed in all samples from the hopper stands out, which was not observed for either ethanol or acetaldehyde. This makes it difficult to draw conclusions about methanol production during short-term storage, as was observed in other research [25,33]. Even so, a certain increase was observed when comparing the reference samples with samples that had passed through the hopper, although it was only statistically significant when all data were considered (Table 3).

Finally, a correlation between the contents of methanol and acetaldehyde (r = +0.50 with *p* = 0.0025) was observed, which is also interesting. This behavior could be due to the fact that methanol, as in other plants, can act as an inhibitor of the enzymatic activity of alcohol dehydrogenase (ADH), stopping the reduction of acetaldehyde to ethanol [32]. This theory would explain that the contents of methanol and acetaldehyde show the same trend in all experiments (Table 3). Moreover, Frenkel et al. [35] also reported that ethanol can act as an inhibitor of the enzymatic activity of pectin methylesterase (PME), and this could be the reason why, in some cases, the amount of methanol is very low when that of ethanol is high (such as in the case of T20 on 12 Dec.). Therefore, the hypothesis that emerges from our study is that the largest increase in ethanol content is due to the degradation of olive fruits and their fermentation after being stored in the hopper. 

**Figure 2:**
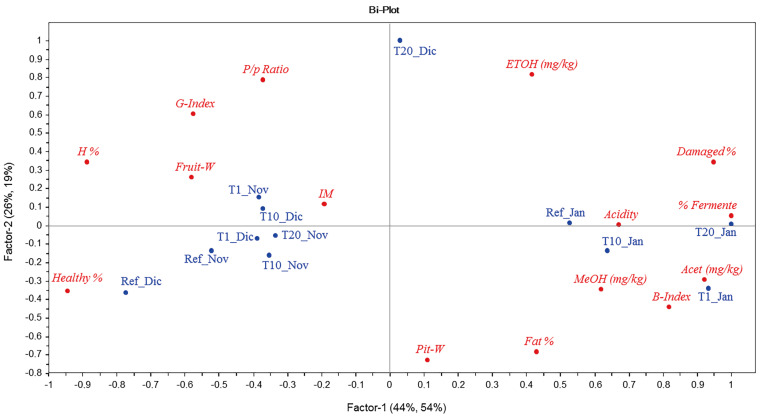
Bi-Plot of the scores (in blue) and loadings (in red) of the PLS regression model using individual data from Table 1, Table 2 and Table 3.

Next, a PLS regression model was built to find relationships between the fruit characteristics and content of alcohols (X predictor variables) and the sensory attributes (Y predicted variables). Figure 2 shows the biplot (joint plot of scores and loadings) for the first two factors of the PLS model. A correlation is observed between the content of alcohols and fruit damage together with the black index [27,34]. In addition, PLS loading highlights the differences between the fruits harvested in November and December (grouped on the negative side of factor 1) from those harvested in January (on the opposite side). The first two groups consist of good-quality fruits, as shows its correlation with a healthy %, while those of January were of lower quality. Further, in each experiment, the samples that did not pass through the hopper (Ref) had better quality and were negatively correlated with the content of alcohols [31].

**Figure 3:**
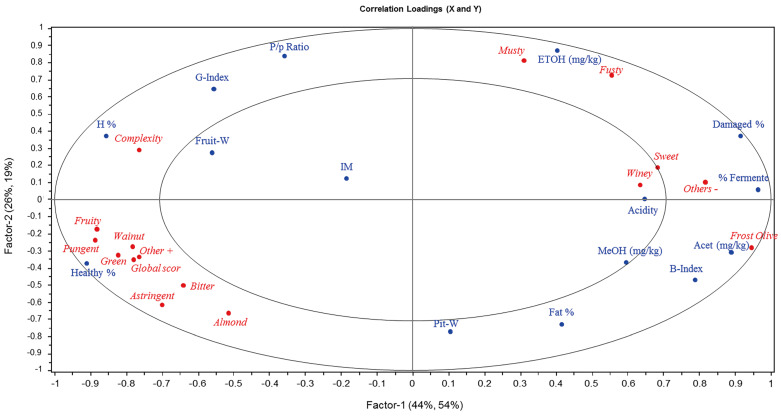
Correlation loadings of the PLS model for the X-variables: fruit characteristics and alcohol content (blue) and Y-variables: sensory attributes (red).

Figure 3 shows the plot of the correlation loadings for the first two factors of the PLS model, that is, the correlations between each X and Y variable and the selected factors. The concentric circles correspond to 50% (inner) and 100% (outer) of explained variance, respectively. The importance of individual variables is visualized more clearly in the correlation loadings plot compared to the standard loadings plot. In our case, Figure 3 shows a high correlation between the content of alcohols and sensory defects. In fact, all the positive attributes, together with the health status of the fruits, are located on the negative side of factor 1, which explains 44% and 54% of the X and Y variance, respectively, whereas all the sensory defects are on the positive side together with alcohols and fruit damages.

Furthermore, ethanol correlates positively with both factors (+0.40 and +0.87 respectively) being the best predictor for the musty and fusty defects and slightly worse for the winey, frost olive, and sweet defects. This makes sense because yeasts that produce ethanol through sugar fermentation may add certain fusty defects to the oil and some others can also cause the degradation of polyphenols, with the consequent decrease in bitterness and increase in sweetness [8,31]. In addition, the musty defect has been related to a complex series of compounds, among which ethanol and hexanal stood out [19]. On the other hand, it has been reported that the winey–vinegary defect is due to an anaerobic fermentation process in olives that leads to the formation of acetic acid, ethyl acetate, and ethanol [15,19,31]. Regarding fruit characteristics, Figure 3 corroborated that, as explained above, the variables % damaged and % fermented show a moderate correlation with ethanol content.

As for acetaldehyde, it is highly correlated with factor 1 (+0.89) and the PLS loading appears to have a trend of the methanol behavior more than that of ethanol. This could be due to the fact that the accumulation of methanol can reduce the chemical reduction of acetaldehyde into ethanol [35]. Moreover, the PLS correlation loadings show that acetaldehyde is related to fruit damage (including % fermented) and black index. This behavior can be explained by the fact that acetaldehyde is generated from the pyruvate pathway and then is reduced to ethanol; in this case, it would be more related to the fruit ripening than to its damage [27,33]. 

Finally, methanol is less explained than ethanol and acetaldehyde by the model, so the results that can be extracted are not conclusive. It correlates positively with the first factor (+0.60) and negatively with the second factor (−0.37), showing a slight correlation with the frost olive and winey defects, although this cannot be fully assured because the number of samples with these defects was low. The best correlation of methanol with fruit characteristics is through the B index. This behavior agrees with the hypothesis that methanol is accumulated during the ripening process of the fruit [23].

## 4. Conclusions

The short-term storage of olive fruits in a large-capacity hopper is a practice widely used in olive oil mills. However, the results obtained in this study show that during this storage the olive fruits suffer a quality deterioration due to the impact of the hopper charge, the high pressure applied by the weight of the olives themselves, and also to the friction with the hopper walls. In addition, it has been detected that the olives located at the bottom or on the sides of the hopper wall suffer a greater impact that accelerates certain microbiological and metabolic activities. These induce a significant increase in the amount of alkyl alcohols, which is directly related to the loss of sensory quality of the oils obtained. Thus, although it is possible to obtain extra virgin olive oil when good quality fruits of ‘Arbequina’ are stored in hoppers for a short term, it is very difficult to achieve a quality premium oil. Therefore, this practice should be reconsidered during the production of high-quality olive oil.

## Figures and Tables

**Table 1:** Mean values of the physicochemical properties of olives from the different sampling dates.

Sample		^(1)^ Healthy (%)	^(1)^ Damaged (%)	Olives T °C	^(2)^ Maturity Index	^(2)^ Fruit Weight (g)	^(2)^ Flesh/Pit Ratio	^(2)^ Moisture (%)	^(2)^ Oil Content (%)
**27 Nov.**	**Ref**	88 a	12 a	9	2.4 b ± 0.2	1.08 b	4.1 b ± 0.3	53.1 a ± 1.2	21.9 a ± 1.6
	**T1**	82 a	18 a	11					
	**T10**	82 a	18 a	12					
	**T20**	80 a	20 a	12					
**12 Dec.**	**Ref**	94 a	6 c	7	2.5 b ± 0.2	1.28 a	5.1 a ± 0.8	54.1 a ± 2.3	21.5 a ± 1.8
	**T1**	76 b	24 b	10					
	**T10**	74 b	26 b	12					
	**T20**	52 c	48 a	12					
**7 Jan.**	**Ref**	60 a	40 a	5	3.5 a ± 0.3	1.03 b	3.4 b ± 0.4	49.4 b ± 1.6	23.7 b ± 1.1
	**T1**	58 a	42 a	6					
	**T10**	58 a	42 a	7					
	**T20**	46 a	54 a	10					

^(1)^ By column, within each time group, means with the same letters are not significantly different according to the X^2^ test (*p* < 0.05); ^(2)^ By column, means with the same letters are not significantly different according to Duncan´s multiple range tests (*p* < 0.05).

**Table 2:** Results of the sensory analysis of the oils obtained from each olive sample.

Attributes		27 Nov.				12 Dec.				7 Jan.			
		Ref	T1	T10	T20	Ref	T1	T10	T20	Ref	T1	T10	T20
negative attributes	Fusty	0.0 a ± 0.0	0.0 a ± 0.8	0.0 a ± 0.0	0.0 a ± 0.0	0.0 b ± 0.0	0.0 b ± 0.0	0.0 b ± 0.0	0.7 a ± 0.6	0.2 a ± 0.0	0.2 a ± 0.0	0.3 a ±0.0	0.3 a ± 0.0
	Musty	0.0 a ± 0.0	0.0 a ± 0.2	0.0 a ± 0.1	0.0 a ± 0.0	0.0 b ± 0.0	0.0 b ± 0.0	0.0 b ± 0.0	1.9 a ± 0.8	0.2 a ± 0.0	0.5 a ± 0.1	0.4 a ± 0.1	0.3 a ± 0.0
	Winey	0.0 a ± 0.0	0.0 a ± 0.3	0.0 a ± 0.1	0.0 a ± 0.0	0.0 b ± 0.0	0.1 ab ± 0.3	0.0 b ± 0.0	0.5 a ± 0.4	0.0 a ± 0.0	0.5 a ± 0.2	0.0 a ± 0.0	0.3 a ± 0.0
	Frost Olive	0.0 a ± 0.0	0.0 a ± 0.0	0.0 a ± 0.0	0.0 a ± 0.0	0.0 a ± 0.0	0.0 a ± 0.0	0.0 a ± 0.0	0.0 a ± 0.0	1.6 a ± 1.1	3.8 a ± 0.7	2.7 a ± 0.4	2.8 a ± 1.4
	Rancid	0.0 a ± 0.0	0.0 a ± 0.0	0.0 a ± 0.0	0.0 a ± 0.0	0.0 a ± 0.0	0.0 a ± 0.0	0.0 a ± 0.0	0.0 a ± 0.0	0.0 a ± 0.0	0.0 a ± 0.0	0.0 a ± 0.0	0.0 a ± 0.0
	Others	0.0 a ± 0.0	0.0 a ± 0.0	0.0 a ± 0.0	0.0 a ± 0.0	0.0 a ± 0.0	0.0 a ± 0.4	0.0 a ± 0.0	0.0 a ± 0.3	0.0 a ± 0.3	0.0 a ± 0.4	0.0 a ± 0.1	0.0 a ± 0.8
positive attributes	Fruity	4.8 a ± 0.2	3.7 b ± 0.4	3.6 a ± 0.4	4.3 ab ± 0.2	5.1 a ± 0.2	4.9 a ± 0.2	4.8 a ± 0.2	3.2 b ± 0.6	3.1 a ± 0.6	2.5 a ± 0.6	2.9 a ± 0.6	2.8 a ± 0.8
	Bitter	3.6 a ± 0.2	2.7 b ± 0.2	3.4 ab ± 0.4	3.6 a ± 0.2	4.2 a ± 0.3	4.2 a ± 0.2	4.3 a ± 0.3	2.1 b ± 0.4	2.1 b ± 0.4	3.2 a ± 0.5	2.7 ab ± 0.3	2.6 ab ± 0.5
	Pungent	4.4 a ± 0.2	4.2 a ± 0.3	4.5 a ± 0.2	4.5 a ± 0.2	4.7 a ± 0.2	4.8 a ± 0.2	4.6 a ± 0.3	3.7 b ± 0.2	3.2 a ± 0.4	3.6 a ± 0.3	3.4 a ± 0.4	3.5 a ± 0.4
	Green	2.7 a ± 0.4	2.0 a ± 0.9	2.3 a ± 0.2	2.4 a ± 0.1	3.7 a ± 0.1	3.7 a ± 0.4	3.2 a ± 0.3	1.2 b ± 0.6	1.2 a ± 0.5	1.1 a ± 0.7	1.1 a ± 0.7	1.3 a ± 0.7
	Sweet	4.3 a ± 0.1	4.6 a ± 0.1	4.0 a ± 0.3	4.4 a ± 0.2	4.3 a ± 0.2	4.2 a ± 0.3	4.1 a ± 0.6	4.7 a ± 0.3	5.1 a ± 0.2	4.6 a ± 0.1	5.1 a ± 0.1	4.8 a ± 0.2
	Astringent	2.2 a ± 0.1	1.1 b ± 0.6	1.8 ab ± 0.4	1.8 ab ± 0.3	2.1 a ± 0.4	2.1 a ± 0.4	1.9 a ± 0.4	0.2 b ± 0.2	0.4 a ± 0.1	1.1 a ± 0.8	0.9 a ± 0.4	0.6 a ± 0.4
	Almond	2.3 a ± 0.2	1.8 a ± 0.4	1.1 a ± 0.7	1.7 a ± 0.6	2.7 a ± 0.1	2.3 a ± 0.2	2.4 a ± 0.4	0.0 b ± 0.5	1.4 a ± 0.4	1.2 a ± 0.8	1.1 a ± 0.7	1.1 a ± 0.8
	Walnut	1.4 a ± 0.8	1.0 a ± 0.9	0.6 a ± 0.2	1.1 a ± 0.8	1.7 a ± 0.3	1.5 a ± 0.8	1.3 a ± 0.7	0.5 a ± 0.1	0.7 a ± 0.4	0.6 a ± 0.2	0.6 a ± 0.2	0.6 a ± 0.2
	Other	2.3 a ± 0.1	2.1 a ± 0.4	1.6 a ± 0.8	1.7 a ± 0.9	2.7 a ± 0.1	2.4 a ± 0.2	2.1 ab ± 0.2	1.4 b ± 0.6	1.6 a ± 0.5	1.3 a ± 0.7	1.7 a ± 0.6	1.6 a ± 0.4
Complexity (*)		4.0	3.0	2.0	2.0	6.0	4.0	4.0	5.0	2.0	1.0	1.0	1.0
Global score (*)		7.2	5.9	6.3	6.4	7.6	6.7	7.2	5.4	5.6	5.3	5.5	5.4

By line and block, means with the same letters are not significantly different according to Duncan´s multiple range tests (*p* < 0.05). (*) Shows a unique value and represents a global evaluation for each column.

**Table 3:** Contents of acetaldehyde (Acet), ethanol (EtOH), and methanol (MeOH) in mg/kg (±SDV) measured in olive samples on different positions (Ref, T1, T10, and T20) during hopper discharging.

Compound	Date	Ref	T1	T10	T20	Average
Acet.	27 Nov.	0.7 a ± 0.1	1.2 ab ± 0.4	1.3 b ± 0.2	1.9 ^C^ ± 0.2	1.26 A
	10 Dec.	0.8 a ± 0.2	1.0 ab ± 0.1	1.2 b ± 0.2	1.9 ^C^ ± 0.1	1.01 A
	7 Jan.	3.1 a ± 0.3	3.3 a ± 0.3	3.0 a ± 0.6	3.0 a ± 0.4	3.10 B
	average	1.76 A	1.76 A	1.78 A	1.94 A	
EtOH	27 Nov.	36.5 a ± 11.2	78.7 b ± 3.6	77.0 b ± 8.3	71.3 b ± 4.5	65.9 A
	10 Dec.	23.7 a ± 9.5	83.6 b ± 6.3	91.8 b ± 4.5	201.3 c ± 19.1	134.3 B
	7 Jan.	138.3 a ± 11.7	106.6 a ± 13.5	118.5 a ± 34.4	121.4 a ± 17.9	122.6 B
	average	71.5 A	87.5 B	95.8 B	164.7 C	
MeOH	27 Nov.	66.1 a ± 40.4	132.9 a ± 52.8	100.8 a ± 33.2	174.2 a ± 50.7	123.3 AB
	10 Dec.	53.2 a ± 4.7	86.6 a ± 71.1	110.1 a ± 52.4	57.5 a ± 36.9	79.0 A
	7 Jan.	100.7 a ± 29.8	232.7 a ± 135.0	126.9 a ± 68.6	176.3 a ± 41.5	159.2 B
	average	77.3 A	150.7 B	112.6 B	136.0 B	

By line and block, means with the same letters are not significantly different according to Duncan´s multiple range tests (*p* < 0.05). The Duncan test for the averages was carried out using the individual data of each block.

## Data Availability

The data presented in this study are available on request from the corresponding author.
